# "Against the silence": Development and first results of a patient survey to assess experiences of safety-related events in hospital

**DOI:** 10.1186/1472-6963-8-59

**Published:** 2008-03-20

**Authors:** David LB Schwappach

**Affiliations:** 1 Research Institute for Public Health and Addiction, Konradstrasse 32, 8031 Zurich, Switzerland; 2 University Witten Herdecke, Faculty of Medicine, Alfred-Herrhausen-Str. 50, 58448 Witten, Germany

## Abstract

**Background:**

Involvement of patients in the detection and prevention of safety related events and medical errors have been widely recommended. However, it has also been questioned whether patients at large are willing and able to identify safety-related events in their care. The aim of this study was to develop and pilot test a brief patient safety survey applicable to inpatient care in Swiss hospitals.

**Methods:**

A survey instrument was developed in an iterative procedure. The instrument asks patients to report whether they have experienced specific undesirable events during their hospital stay. The preliminary version was developed together with experts and tested in focus groups with patients. The adapted survey instrument was pilot-tested in random samples of patients of two Swiss hospitals (n = 400). Responders to the survey that had reported experience of any incident were sampled for qualitative interviews (n = 18). Based on the interview, the researcher classified the reported incidents as confirmed or discarded.

**Results:**

The survey was generally well accepted in the focus groups and interviews. In the quantitative pilot test, 125 patients returned the survey (response rate: 31%). The mean age of responders was 55 years (range 17–91, SD 18 years) and 62.5% were female. The 125 participating patients reported 94 "definitive" and 34 "uncertain" events. 14% of the patients rated any of the experienced events as "serious". The definitive and uncertain events reported with highest frequency were phlebitis, missing hand hygiene, allergic drug reaction, unavailability of documents, and infection. 23% of patients reported some or serious concerns about their safety. The qualitative interviews indicate that both, the extent of patients' uncertainty in the classification of events and the likelihood of confirmation by the interviewer vary very much by type of incident. Unexpectedly, many patients reported problems and incidents related to food and dietary intake. Overall, the in-depth interviews confirmed experiences from the focus groups that many patients feel reluctant to report undesirable events without acknowledging the presence or absence of individual responsibility or failure. Many patients reported that they did not ask or communicate about errors or near misses with staff and some patients even develop strategies to improve their safety but do not disclose these to staff.

**Conclusion:**

Many patients experience undesirable events during hospitalization and a significant number of patients is seriously concerned about their safety. Surveying patients about experiences with safety-related events in hospital seems a valuable tool for identifying and monitoring problematic areas of care and undesirable events. Evidence from the qualitative interviews indicates that safety remains an unsaid word between patients and their care providers.

## Background

Involvement of patients in the detection and prevention of safety related events and medical errors has been widely recommended, e.g., by the Institute of Medicine (IOM), the American Hospital Association as well as academic and clinical experts [1-4]. Since patients are the only individuals physically present during every treatment and consultation, they carry with them important contextualized information as they move through a complex and distributed process of care and are thus a valuable resource for safe and effective treatment systems [5]. As Unruh und Pratt describe from a series of case studies, patients often engage in a range of tasks to identify and intercept errors and much of patients' work revolves around integrating and communicating health information between different organizational units in which they receive care. Thus, surveying patients about adverse events experiences during their hospital stay may yield information otherwise not available. Few empirical studies have recently been conducted that addressed patients' experiences with adverse events or medical errors and report encouraging results [6-12]. For example, Weingart et al. elicited incident reports from hospital inpatients in order to identify and characterize adverse events and near misses [6]. Safety-related problems were identified by medical record review and patient interviews followed by physician classification of patients' reports. In this study, 228 participating patients experienced 20 adverse events and 13 near misses. Of these, only 55% of adverse events and 31% of near misses were documented in the medical record, and none were found in the hospital incident reporting system. While it has been well-recognized that patient reporting of adverse events may be contaminated by false-positive and false-negative incidents [7], these results also suggest that errors detected by patients are not easily identified by other means. The assessment of patient-reported safety-related events may thus add to a broader understanding of safety related problems in inpatient care and supplement other monitoring approaches, e.g., critical incident reporting systems, staff surveys and evaluation of safety culture [13-15]. However, it has also been questioned whether patients at large are willing and able to identify safety-related events in their care [16]. For example, patients may be unaware of, or misinterpret undesirable events, may lack opportunities to observe failures, and may feel reluctant to question professionals' actions [17]. The aim of this study was to extend on prior work and develop and pilot test a brief patient safety survey applicable to inpatient care in Swiss hospitals. This survey should return data on specific events and experiences and thereby provide direct input for in-depth analysis of safety concerns or for improvement activities. We used a concurrent mixed-methods approach, i.e., a combination of qualitative and quantitative methods to characterize incidents reported by patients and validate their nature by assessing the event, or "story", behind survey responses.

## Methods

This study comprised three phases: In phase I, a preliminary version of the survey was developed in an iterative procedure, compromising a literature review and discussion with 8 national and international experts. The survey included a core question that asks for experience of a number of safety-related, undesirable events. This item list was based on a survey developed by Agoritsas et al. [7]. The term "undesirable event" broadly describes suboptimal outcomes and processes that may or may not be resulting from error, and may or may not result in consecutive harm. The original item list used by Agoritsas et al. included 27 items (9 medical complications, 9 interpersonal problems, and 9 incidents related to the care process). This list was revised according to a number of criteria: First, interpersonal problems were dropped from the list since the majority of Swiss hospitals routinely utilize patient surveys for outcome measurement and the assessment of patient satisfaction, and it had to be ensured that any redundancies with common instruments in use are avoided [18-20]. Thus, certain themes that are also important from a safety perspective, e.g., appropriate communication, were omitted by intent. Second, the list of medical incidents and process problems was adjusted. New items were included and existing items were excluded from the original list. This adjustment process was based on various criteria, including expected incidences of events and noticeability by patients. For example, the original item list did not include items on medication errors and missing hand hygiene, both important and common safety-related events that were suggested by experts. Other items, such as reoperation, intensive care transfer, or reactions to blood transfusions were excluded from the list, mainly because low incidences and the fact that these items are commonly measured in routine outcome measurement (e.g., surgical complications). Five new survey questions were developed included ratings of the severity of the self-reported events, communication with staff about events and concerns for safety. The survey asked patients to report their personal experiences and ratings of care and choose those answers that would meet their personal views best.

The preliminary survey was tested in two focus groups with hospitalized patients aged 47–80 years (median age: 72). Patients were asked to complete the survey on their own during quiet time while a researcher recorded the time needed to complete and observed participants' behaviour and noted any obvious confusion or questions that arose. After completion, patients were asked to think-aloud retrospectively, i.e., verbalize their thoughts and describe how they arrived at their answers. They then discussed any problems they encountered with the survey, whether any questions were hard to understand or to respond to, and whether they felt that others may have problems with responding to the survey. Focus group meetings were digitally recorded and transcribed. Based on the results of the focus groups, the survey was adapted in wording and content. For example, the original item list by Agoritsas et al. only asked patients whether they experienced any of the events. In the focus groups in our study, many patients were reluctant to provide yes-no answers to the item list. These patients argued that in some instances, they were uncertain with their observation and they did not want to provide "wrong" feedback on their provider. Therefore, a distinction between "uncertain" and "definitive" events was introduced to reflect patients' concerns to indicate levels of uncertainty in judgment. Thus, in the adapted version, for each event patients were asked to report whether the event occurred and were provided the response categories "yes", "no" and "possibly". Many items were revised in wording, mainly because patients did not understand the original item list (the majority of revisions) or in few cases due to theoretical considerations. As an example for the former, the original item that asked whether patients had experienced an infection during hospital stay was revised to include examples of common infections (urinary tract infection, wound infection, etc.) as in the focus groups some patients inquired about the term "infection". Also, the item asking for experience of phlebitis was reworded with two clinicians to describe the event in lay terms ("aching redness of a vein"). As an example for modifications due to theoretical considerations, the original item relating to in-hospital falls asked patients whether they were *injured *in a fall. This was reduced to any experience of falls to maintain coherency with other items that also do not include dimensions of harm, e.g., medication errors, and because falls in general pose the risk for serious harm and need therefore to be avoided.

In phase II, the adapted survey instrument was pilot-tested in samples of patients of two Swiss hospitals, one large teaching hospital (860 beds) and a community hospital (170 beds). Patients were randomly sampled from general surgical and internal medicine wards. The only inclusion criteria were a minimum of two days of prior stay in the hospital and the ability to read and understand German language. The questionnaires were administered to patients during their inpatient stay personally by research assistants together with a pre-paid envelope. The survey was accompanied by an explanatory letter signed by the research institution and the respective hospital director. The survey was introduced by the following statement: "Staff in this hospital works with high dedication and diligence to improve patients' health and to avoid any harm. Nevertheless, complications, undesirable events and errors may occur during hospitalization. The following survey is part of a research study that assesses safety and quality in hospital care. In this survey, we will ask you whether you experienced any problems, errors, or complications in your care. Results of the study may help to improve hospital care." In the pilot-version of the survey, patients were also asked to provide contact details and written consent in case they were willing to participate in an interview to discuss their survey responses. In phase III, we sampled patients that had returned the survey and reported experience of any incident, agreed to be contacted and provided valid contact details. These patients were approached and interviewed by phone. Interviews were conducted by a trained researcher with a background in sociology and many years of experience in qualitative methods in health services research who had also conducted the focus groups. For all questions and undesirable event items a distinct set of open questions was prepared and combined in an individual, personalized interview framework for each patient depending on her responses in the written survey. This framework mainly addressed details of the reported events (e.g., what type of drugs were involved in case patients had reported an allergic reaction), how patients discovered that something had gone wrong, and whether the incident caused any harm or other consequences. Patients were also interviewed regarding communication with staff and their reports to an open survey question which asked them to describe any other safety-related events they had observed during their stay. Subsequent to each interview, the researcher classified each discussed event as "yes" (confirming that the reported event corresponds to the requested survey item), "no" (verifying that the reported event probably does *not *correspond to the requested survey item) or as "unclear" (confirming that, based on the available information, the reported event was ambiguous). Tape recordings of five interviews were reassessed by the principal investigator and classifications of all events discussed in these interviews were confirmed. It should be noted though that this judgment was entirely based on the additional information provided by patients. Because the study carried minimal risk, it was approved in minimal risk review and exempted from full formal evaluation. All subjects provided written informed consent, which was reconfirmed verbally in subjects participating in qualitative interviews.

## Results

The survey was generally well accepted in the focus groups and patients emphasized the importance of the subject, their interest and their willingness to contribute. Participants needed on average 11 minutes to complete the survey. It became obvious in the group discussions that patients had difficulties in naming and reporting incidents without thinking in terms of causation, preventability, fault and blame. In particular, participants had the strong need to defend their attending physicians from "misleading attribution". For example, while discussing the survey item "experience of in-hospital falls", patients repeatedly argued that it would make an important difference for them whether the fall had been initially caused by an act of negligence of medical staff or by carelessness of hospital cleaners. Another problem encountered in the focus groups was patients' reservation to report minor events and incidents they felt that had not been reconfirmed by staff. As has been outlined, the survey was adapted to meet these needs, e.g., by allowing different grades of certainty in responses to the list of items.

In the quantitative pilot test, 125 of the 400 surveys distributed to patients currently hospitalized in either of the two participating hospitals were returned (31% response rate). The mean age of responders was 55 years (range 17–91, SD 18 years). 62.5% were female. The median time between first day of hospitalization and survey completion was 12 days (mean 20, range 4–102 days). 42% of patients responded to the survey during their hospital stay while 58% answered it after returning home. 95% responded after at least 5 days of hospitalization. The 125 participating patients reported 94 "definitive" events and 34 "uncertain" events. The event rate was thus 0.75 (95%CI 0.54–0.97) for definitive, 0.27 (95%CI 0.15–0.40) for uncertain and 1.02 (95%CI 0.76–1.29) for all events. Reported definitive and uncertain events concentrated in 56 individual patients. Table 1 presents the frequency of reported events. Neither age nor gender were significant predictors of reporting any definitive event. However, the odds of reporting such events rose with every additional day between hospitalization and survey completion (OR = 1.03, 95%CI 1.01–1.05, p = 0.012). 14% of patients that reported at least one undesirable event judged any of the experienced events as "serious". Of all responders, 4% were seriously concerned and 19% reported "some concern" about their safety and errors in their care during hospitalization. Patients that reported at least one definite event during their hospital stay were five times more likely to be "somewhat" or "seriously" concerned about their safety (OR = 5.85, 95%CI 2.39–14.30, p = 0.0001). Communication with hospital staff about this event was reported by 37% of patients that had experienced at least one definitive or unclear event. 14% answered that staff did not communicate about the event and 49% responded that such communication was not necessary. The vast majority perceived communication as (rather) "honest and open" (86%) while the remaining disagreed and rated communication as not or not at all honest.

**Table 1:** Frequencies of safety-related undesirable events reported by patients (n = 125 patients)

		N (%) of survey responses	
		
Nr.	Item	Definitely	Not sure
1	You developed an inflammation or aching redness of a vein (phlebitis) because of an intravenous line.	20 (16)	1 (1)
2	You acquired an infection in the hospital (e.g., urinary tract infection, sepsis, wound infection).	9 (7)	1 (1)
3	You discovered that staff did not disinfect their hands before touching you.	10 (8)	10 (8)
4	You experienced an allergic reaction to a drug.	15 (13)	3 (3)
	Was the hospital informed about the allergy prior to dispensing the drug to you?^a^	5 (28)	1 (6)
5	You were given an infusion or drug that was not intended for you.	1 (1)	--
	You were given an infusion or drug...		
6	at the wrong time, or	5 (4)	--
7	at the wrong dose, or	5 (5)	1 (1)
8	a dose was omitted, by mistake.	6 (5)	3 (3)
9	Your medical record or radiograms were not available when needed.	5 (4)	6 (5)
10	A test was repeated needlessly, by mistake.	3 (2)	2 (2)
11	A planned test was omitted, by mistake.	6 (5)	4 (3)
12	A test, surgical intervention or therapy was nearly or in fact performed on the wrong site of your body.	2 (2)	1 (1)
13	You were confused with another patient during a test or treatment.	2 (2)	1 (1)
14	You experienced a fall in hospital.	5 (4)	1 (1)

^a ^Numbers relate to patients that reported the event either as "definitive" or "uncertain"

Of the 50 survey responders that reported at least one definitive undesirable event during hospital stay, 18 were interviewed by a qualitative researcher. The in-depth interviews took around 30 minutes. The interviewed subjects had reported 40 events in the written survey (30 definite events and 10 uncertain events). Table 2 reports agreement between incidents reported by patients in the written questionnaire and the interviewer's judgment after the interview. Overall, there was a moderate degree of confirmation of the events by the interviewer. 18 of the definite events were confirmed after the interview, 9 were discarded and 3 could not be classified. Of the 10 uncertain events, 8 could not be classified even after the interview, one was discarded and one was confirmed as the described incident. As Table 2 shows, types of events differ in both, the extent of patients' uncertainty in the classification of events and the likelihood of confirmation by the interviewer. For example, 5 out of the 6 responses regarding missing hand hygiene were "uncertain events" and could not be verified by the interviewer. When asked, these patients reported that staff did not disinfect their hands at the bedside prior to physical contact but they did not know whether staff used hand antisepsis on the floor. Some patients also reported that ward rounds involved many individual physicians, including medical students, and they could simply not follow whether, and who did and did not use hand antisepsis before touching them. Patients' survey responses regarding the experience of phlebitis were characterized by a high number of false-positive responses (50% of definitive events discarded by the interviewer). In the majority of these cases, patients had experienced difficult insertion of needles and cannulas, resulting in haematoma, aching, or pain. However, half of the patients reported a diagnosis of phlebitis, and experienced local heat and swelling, fever or were prescribed antibiotics. From patients' explanations of their survey ratings of event severity it became clear that even in case of serious incidents patients' perception of severity depends on *actual *outcomes rather than *potential *for harm. Patients commonly argued that "... though this (event) was a bad experience, everything came to a good end finally".

**Table 2:** Concordance between survey responses and interviewer classification of undesirable events (n = 18 patients)

Item	Nr of survey responses		Interviewer Classification
		
	Definitely	Possibly	
1 – Phlebitis	5		Yes
	5		No
			Unclear
2 – Infection			Yes
	1		No
		1	Unclear
3 – Hand Hygiene			Yes
	1		No
		5	Unclear
4 – Allergic reaction	2		Yes
	1		No
	2		Unclear
6 – Drug – wrong time	5		Yes
			No
			Unclear
8 – Drug – dose omission	2		Yes
			No
			Unclear
9 – Documents n/a	1		Yes
	1	1	No
		1	Unclear
10 – Test repetition			Yes
			No
	1	1	Unclear
11 – Test omission	2		Yes
			No
			Unclear
13 – Patients confused		1	Yes
			No
			Unclear
14 – Fall	1		Yes
			No
			Unclear

The interviews also detailed any other reports provided by patients, either in the written survey or mentioned in the personal interview. Unexpectedly, many patients reported problems and incidents related to food and dietary intake. These involved confusion of special meals, supply of wrong meals, or at the wrong time, and unavailability of meals when needed. Also, problems that affected medication and meals were reported. For example, patients were commonly advised by medical staff to take their oral medication with the meals but drugs and meals were not delivered at due time. Some patients reported that they informed nursing staff about the obvious disconcordance and had then been told to take medications without food. One patient reported that she refused to because she had experienced serious gastrointestinal side effects before, and asked for some small food to take her medication. She had to wait several hours until she could take her medication. Other reports included patients that needed special meals due to allergies or food intolerance (e.g., gluten free diet) and were supplied the normal meal, or the special meal was confused with other patients' meals in the same room.

The interviews disclosed that some patients have distinct strategies to deal with the experience of error or their safety concerns: For example, one female patient reported that she "surreptitiously" disinfected her hands and wound before and after ward rounds herself because she recognized missing hand hygiene. A patient with diabetes said that he did not trust drug preparations and always double-checked medications. However, these, and several other reports clearly showed that patients developed these strategies by themselves and did not want to disclose them to their care provider as they felt this would question medical authority. Many patients reported that they did not ask or communicate about errors or near misses with staff. Two common patterns of explanation for this were observed: Some patients felt uncomfortable with the situation and expressed fears that staff would not welcome their enquiries. For example, a female patient argued that "You just try to care for yourself. You'd better not ask them [the doctors]. You do not want to be a troublemaker. You never know, they may feel controlled or something and then...things may even get worse.". Others did not discuss undesirable events either because the event had no serious consequences or because they did not want to trouble staff. Overall, the in-depth interviews confirmed experiences from the focus groups that many patients feel reluctant to report undesirable events without acknowledging the presence or absence of individual responsibility or failure. Notably, when asked whether the experienced events affected their satisfaction with the hospital stay, virtually all patients disagreed and stated that they would again choose the same hospital.

## Discussion

In this study we report the development and first results of a patient survey to assess patients' experiences with safety-related events in hospitals. The instrument was pilot-tested in two Swiss hospitals and incidents reported by patients were analyzed in-depth based on qualitative interviews with patients. 23% of patients reported some or serious concerns about their safety, a figure very close to results from an Australian survey study in which 5% of patients reported that they would feel very unsafe if admitted to hospital and 20% stated they would feel a little unsafe [21].

The 125 participating patients reported 94 "definitive" and 34 "uncertain" events. 14% of the patients rated any of the experienced events as "serious". The definitive and uncertain events reported with highest frequency were phlebitis, missing hand hygiene, allergic drug reaction, unavailability of documents, and infection. Using the similar, original list of specific events, Agoritsas, Bovier and Perneger estimated the frequency of specific undesirable events reported by adult patients recently discharged from a large university hospital in Switzerland [7]. In this study, every second patient reported at least one out 27 medical, interpersonal and process related undesirable events. The most frequent events were phlebitis (11%), unavailable medical record (9%), failure to respect confidentiality (8%), and hospital-acquired infection (8%). While the two studies differ in various respects, including sample, sample size and response rate, the absolute and relative frequencies of events are very similar. Our study also confirms Agoritsas et al. findings that experience of undesirable medical events is not closely related to satisfaction with care. In their study, patients' satisfaction was correlated with their experience of interpersonal events and process problems, but not medical complications. This suggests that experience of errors and perception of safety and patient satisfaction on the other side are distinct concepts and results of patient satisfaction surveys are no substitute for patients' views on safety.

Our results regarding patients' ability to recognize the specified events are generally positive in that many patients are able to identify specific undesirable events in their care. However, as the qualitative interviews indicate, both, the extent of patients' uncertainty in the classification of events and the likelihood of confirmation by the interviewer vary very much by type of incident. For example, 5 out 10 uncertain events reported by patients and 5 out 11 reports that could neither be confirmed nor discarded by the interviewer related to missing hand hygiene. Here, however, the missing opportunity to observe the action is per definition a confirmation of the event since international guidelines and the national "clean care" campaign recommend hand antisepsis *at the bedside *prior to any physical contact. Other indicators, in particular the question relating to phlebitis, seem more contaminated by uncertainty in observation and judgment. While this questions the use of the survey results "as is", i.e., estimators of incidence, it also highlights their value as indicators of undesirable experiences. In the qualitative interviews, all patients, including those whose reports could not be confirmed as descriptions of phlebitis, substantiated the negative, often painful and irritating, incident. As reported by Weingart et al., who investigated patients' ability to recognize medical errors in an interview study with adults treated in an outpatient oncology infusion unit, many of the incidents detected by the patients in our study can be classified as quality problems rather than errors or safety-related events [8]. However, the limits between quality and safety are often fluent: Confusion of patients' meals may predominantly be a quality problem, however, if patients with food intolerance or allergies are involved, this becomes a real safety concern. The various reports relating to problems with meals that were obtained in the qualitative interviews warrant further investigation. As these reports included a variety of experiences, it was not possible to generate additional survey items "at hand" without prior qualitative testing.

The major limitations of the study are the small sample size and low response rate. While response rates far below 50% are not uncommon in patient surveys distributed during stay and without reminder, it is certainly unsatisfactory and leaves large room for bias [22-24]. Perneger et al. showed in an analysis of nonresponse bias in a survey assessing patients' perceptions of care that the tendency to participate was negatively associated with the report of problems during hospitalization. However, in their study, increasing participation from 30% to 70% had only a modest influence on the final conclusions of the survey [25]. Another problem inherent in the approach to survey patients about undesirable events during hospitalisation and our "validation" interview is that the method helps to identify false-positive, but not false-negative reports. Thus, even among responders, problems may have gone undetected. In addition, patient surveys do not cover those that experienced the most harmful incidents, such as persistent disability or even death. However, "selectivity" has also been shown to affect other approaches to incident reporting that rely on professionals' observation [6,15]. Thus, these different methods should be seen as complements, not substitutes to each other and patients' reports of safety-related events of their hospital stay may return information on problem areas otherwise not available. More generally, we did not validate patients' reports against more objective measures of incidents or investigated traditional measures of reliability. While an investigation of agreement with other methods of incident reporting would be valuable, it is unlikely that comparison with one single data source would suffice. For example, while indicators of nosocomial infections are probably well documented in medical records, there is little data available ad hoc to assess hand hygiene or confusion of patients. For each of these items, different methods of validation may be needed, e.g. interviews with staff, correlation of reports of missing hand hygiene with infection rates, or even quantities of used disinfectants. In this study, we had used qualitative interviews to assess the reported events in context. The results obtained show a more comprehensive picture of patients' perceptions of undesirable events, limits to noticeability, and the processes surrounding these events. The approach therefore targets patients' transformation of complex, subjective experiences, perceptions and judgments into single item survey questions. The data obtained clearly helps to verify and interpret the quantitative findings. However, the approach has its limits. Even after the interviews, there remains a certain degree of uncertainty and the nature of a number of events could not be determined unequivocally. It should be noted though, that the interview study helped to identify and clarify on the two events most strongly associated with uncertainty (missing hand hygiene and phlebitis), and also the reasons underlying ambiguity in observation and judgment.

Other researchers have reported that patients feel uncomfortable with acting as "vigilant partners" and participating in error prevention if this involves questioning medical authority, such as asking professionals to wash their wands or confirming the right medication and dose [26]. However, Hibbard et al. also show that an individual's perceived self-efficacy, i.e., how efficacious one feels in the ability to prevent errors, is strongly related to the reported likelihood of taking preventive actions. Notably, neither gender, age, education nor self-rated health status were predictors of self-efficacy but having heard about medical errors in the past and the number of nights a family member stayed in hospital in the past year were significant correlates of self-efficacy. Our study adds that many patients do not communicate with staff about their safety concerns and some patients even develop strategies to increase their own safety, but do not want their providers to know. These results, together with the observation that patients have problems with naming incidents without thinking in terms of fault or negligent behaviour indicates that there is still big silence between patients and their providers, and safety often remains an unsaid word. If patients are to be involved in error detection or prevention, they need to be seriously motivated by professionals and invited to ask and report their experiences. This survey instrument may be a valuable tool to monitor hospitals' efforts and success in doing so.

## Conclusion

Surveying patients about experiences with safety-related events in hospital seems a promising approach and valuable tool for identifying and monitoring problematic areas of care and undesirable events during hospitalization. Future studies need to explore the value of the results obtained for quality improvement activities. Patients often have problems to name incidents without thinking in terms of fault and blame and sometimes even seek their private strategies to ensure their safety. The results suggest that safety is still a tabooed ground within the patient-provider relationship.

## Competing interests

The author(s) declare that they have no competing interests.

## Authors' contributions

DLBS designed and conducted this study and prepared the manuscript.

## Pre-publication history

The pre-publication history for this paper can be accessed here:



## References

[B1] Kloth DD (2002). Prevention of Chemotherapy Medication Errors. Journal of Pharmacy Practice.

[B2] Institute of Medicine (2000). To err is human Building a safer health system.

[B3] Vincent CA, Coulter A (2002). Patient safety: what about the patient?. Qual Saf Health Care.

[B4] Coulter A (2006). Patient safety: what role can patients play?. Health Expect.

[B5] Unruh KT, Pratt W (2006). Patients as actors: The patient's role in detecting, preventing, and recovering from medical errors. Int J Med Inform.

[B6] Weingart SN, Pagovich O, Sands DZ, Li JM, Aronson MD, Davis RB, Bates DW, Phillips RS (2005). What can hospitalized patients tell us about adverse events? Learning from patient-reported incidents. J Gen Intern Med.

[B7] Agoritsas T, Bovier PA, Perneger TV (2005). Patient reports of undesirable events during hospitalization. J Gen Intern Med.

[B8] Weingart SN, Price J, Duncombe D, Connor M, Sommer K, Conley KA, Bierer BE, Ponte PR (2007). Patient-Reported Safety and Quality of Care in Outpatient Oncology. Joint Commission Journal on Quality and Patient Safety.

[B9] Burroughs TE, Waterman AD, Gallagher TH, Waterman B, Adams D, Jeffe DB, Dunagan WC, Garbutt J, Cohen MM, Cira J (2005). Patient concerns about medical errors in emergency departments. Acad Emerg Med.

[B10] Burroughs TE, Waterman AD, Gallagher TH, Waterman B, Jeffe DB, Dunagan WC, Garbutt J, Cohen MM, Cira J, Fraser VJ (2007). Medication Safety: Patients' Concerns About Medical Errors During Hospitalization. Joint Commission Journal on Quality and Patient Safety.

[B11] Wasson JH, MacKenzie TA, Hall M (2007). Patients use an internet technology to report when things go wrong. Qual Saf Health Care.

[B12] MacPherson H, Scullion A, Thomas KJ, Walters S (2004). Patient reports of adverse events associated with acupuncture treatment: a prospective national survey. Qual Saf Health Care.

[B13] Murff HJ, Patel VL, Hripcsak G, Bates DW (2003). Detecting adverse events for patient safety research: a review of current methodologies. J Biomed Inform.

[B14] Miller MR, Elixhauser A, Zhan C, Meyer GS (2001). Patient Safety Indicators: using administrative data to identify potential patient safety concerns. Health Serv Res.

[B15] Olsen S, Neale G, Schwab K, Psaila B, Patel T, Chapman EJ, Vincent C (2007). Hospital staff should use more than one method to detect adverse events and potential adverse events: incident reporting, pharmacist surveillance and local real-time record review may all have a place. Qual Saf Health Care.

[B16] Lyons M (2007). Should patients have a role in patient safety? A safety engineering view. Qual Saf Health Care.

[B17] Davis RE, Jacklin R, Sevdalis N, Vincent CA (2007). Patient involvement in patient safety: what factors influence patient participation and engagement?. Health Expect.

[B18] Schwappach D, Blaudszun A, Conen D, Eichler K, Hochreutener MA, Koeck C (2004). Women's experiences with low-risk singleton in-hospital delivery in Switzerland. Swiss Med Weekly.

[B19] Schwappach DL, Blaudszun A, Conen D, Ebner H, Eichler K, Hochreutener MA (2003). 'Emerge': benchmarking of clinical performance and patients' experiences with emergency care in Switzerland. Int J Qual Health Care.

[B20] Schwappach DLB, Hochreutener M-A, Conen D, Eichler K, Blaudszun A, Koeck CM (2003). Outcome measurement in the Canton of Zurich From theory to practice – Progress report on six years of development, implementation and experience with routine inpatient outcome measurement.

[B21] Evans SM, Berry JG, Smith BJ, Esterman AJ (2006). Consumer perceptions of safety in hospitals. BMC Public Health.

[B22] Sitzia J, Wood N (1998). Response rate in patient satisfaction research: an analysis of 210 published studies. Int J Qual Health Care.

[B23] Castle NG, Brown J, Hepner KA, Hays RD (2005). Review of the literature on survey instruments used to collect data on hospital patients' perceptions of care. Health Serv Res.

[B24] Gasquet I, Falissard B, Ravaud P (2001). Impact of reminders and method of questionnaire distribution on patient response to mail-back satisfaction survey. J Clin Epidemiol.

[B25] Perneger TV, Chamot E, Bovier PA (2005). Nonresponse bias in a survey of patient perceptions of hospital care. Med Care.

[B26] Hibbard JH, Peters E, Slovic P, Tusler M (2005). Can patients be part of the solution? Views on their role in preventing medical errors. Med Care Res Rev.

